# High Prevalence of Cholelithiasis in Umbilical Hernia Patients and Vice Versa: A Prospective Multicenter Study

**DOI:** 10.7759/cureus.85900

**Published:** 2025-06-13

**Authors:** Hakan Kulacoglu, Alpaslan Sahin, Celil Ugurlu, Haydar Celasin, Kayihan Akin

**Affiliations:** 1 Department of General Surgery, Ankara Hernia Center, Ankara, TUR; 2 Department of General Surgery, University of Health Science, Hamidiye School of Medicine, Konya City Hospital, Konya, TUR; 3 Department of General Surgery, Tokat Gaziosmanpaşa University School of Medicine, Tokat, TUR; 4 Department of General Surgery, Lokman Hekim University School of Medicine, Ankara, TUR; 5 Department of Radiology, Lokman Hekim University School of Medicine, Ankara, TUR

**Keywords:** cholelithiazis, high bmi, obesity, risk factors, umbilical hernia

## Abstract

Background

Cholelithiasis (CL) and umbilical hernia (UH) often coexist, but their exact relationship remains underexplored. Both conditions share common risk factors such as obesity, female gender, and metabolic disorders, suggesting a potential pathophysiological link. Despite numerous retrospective analyses, robust prospective multicenter data are lacking. This study aims to provide a more accurate estimation of the prevalence of UH in CL patients and vice versa, identify independent risk factors, and assess the predictive value of BMI in this association.

Methods

In this prospective, multicenter, cross-sectional study, 278 adult patients presenting with either CL or UH were enrolled across four tertiary medical centers between October 2023 and September 2024. Participants were categorized into CL and UH groups based on clinical and radiological assessments. Demographics, clinical characteristics, and comorbidities were carefully analyzed. Multivariate logistic regression identified independent risk factors, while receiver operating characteristic (ROC) curve analysis determined optimal BMI thresholds predictive of CL and UH.

Results

A total of 44 (25.9%) patients with CL had UH, while 32 (29.6%) patients with UH had CL. Multivariate analysis revealed female gender (OR: 0.221, 95% CI: 0.068-0.724, p = 0.013) and obesity (BMI ≥ 30 kg/m², OR: 3.443, 95% CI: 1.337-8.886, p = 0.001) as significant independent risk factors. ROC analysis identified a BMI cut-off of 26.5 kg/m² for predicting CL in UH patients (AUC: 0.679, p = 0.001) and 28.8 kg/m² for predicting UH in CL patients (AUC: 0.748, p < 0.001).

Conclusion

The observed co-prevalence of CL and UH suggests a potential association that may warrant further clinical attention in carefully evaluated cases. Ultrasound screening for cholelithiasis may be considered in umbilical hernia patients with elevated BMI or other risk factors, as it may assist in surgical planning and help avoid intraoperative difficulties related to adhesions during possible future cholecystectomy. These findings suggest a potentially meaningful clinical association and support the need for further investigation into shared pathophysiological mechanisms.

## Introduction

Umbilical hernia (UH), though less common than inguinal hernia, remains a surgical challenge due to its relatively high recurrence rate, even with mesh reinforcement [1,2]. Cholecystectomy, on the other hand, is one of the most commonly performed operations, with rates comparable to inguinal hernia repair in many Western countries [3-5]. Gallstone prevalence in Western populations ranges from 10% to 20%, with known risk factors including obesity, female sex, advanced age, and a history of childbirth [6,7]. Similarly, UH is associated with obesity, multiparity, and other conditions linked to connective tissue disorders [8].

The increasing use of imaging and the widespread adoption of laparoscopic cholecystectomy have led to a marked rise in the detection and surgical management of gallstones [9,10]. While some studies have suggested a possible link between cholelithiasis (CL) and UH, most are retrospective, limiting accurate estimation of prevalence and risk factors [11,12]. The lack of prospective, multicenter data leaves important questions unanswered.

This study aims to provide a prospective, multicenter analysis to better characterize the coexistence of CL and UH and to evaluate whether a clinically relevant association exists between them.

This study was presented as a conference abstract at the 46th European Hernia Society Annual Conference, May 29-31, 2024, Prague, Czech Republic [13]. 

## Materials and methods

Trial design

This prospective, multicenter, cross-sectional study was approved by the Local Ethics Committee (approval date/number: 2023-16-13) and registered on ClinicalTrials.gov (NCT06522308). The study was conducted in accordance with the principles of the Declaration of Helsinki. Written informed consent was obtained from all participants prior to enrollment. To ensure clarity, transparency, and reproducibility, the study was designed in accordance with Strengthening the Reporting of Observational Studies in Epidemiology (STROBE) guidelines. It was carried out across three university hospitals and one specialized hernia center in Turkey.

Participants and eligibility criteria

A total of 278 patients aged 18 years or older who presented electively to the general surgery outpatient clinics of the participating centers between October 2023 and September 2024 were evaluated for inclusion. Eligibility criteria excluded individuals with a history of cholecystectomy, umbilical hernia repair, or incisional hernia repair. Additional exclusion criteria included previous surgical interventions involving the gallbladder or abdominal wall hernias, advanced liver disease, active infection or systemic inflammatory disorders, pregnancy, or inconclusive ultrasound findings that precluded definitive classification of CL or UH.

Sample size

The sample size was based on the estimated prevalence of CL and UH in the general population. CL affects approximately 10-20% of individuals, with higher rates observed among women and individuals with obesity (BMI ≥30 kg/m²) [6,7,14]. The reported prevalence of UH ranges from 8.7% to 25%, depending on population characteristics and the diagnostic method, with ultrasound detecting more cases than physical examination [15,16].

Over a 12-month enrollment period across four centers, a total of 278 patients were included. This sample size was considered adequate to reflect population-based prevalence patterns and to support subgroup comparisons, multivariable analyses, and receiver operating characteristic (ROC) evaluation.

Group assignment

Patients presenting with clinical symptoms suggestive of CL, including right upper quadrant pain, postprandial discomfort, nausea, and bloating, underwent biliary ultrasound. Those with a confirmed diagnosis of cholelithiasis were assigned to the CL group. All patients in this group were additionally screened for the presence of UH using abdominal ultrasound, even if they had no symptoms of hernia. UH was defined according to the European Hernia Society Classification for primary and incisional abdominal wall hernias as a defect occurring between 3 cm above and 3 cm below the umbilicus. Similarly, patients presenting with UH were first allocated to the UH group and subsequently screened for biliary pathology using abdominal ultrasound to determine the presence of CL.

Clinical and demographic data collection

Demographic and clinical data were collected to evaluate risk factors for the coexistence of CL and UH. Parameters included age, sex, body mass index (BMI), family history of cholelithiasis or hernia, comorbidities (diabetes mellitus, metabolic syndrome, hypertension), tobacco use, and obstetric history.

Main outcomes

Primary outcomes were the prevalence of CL in patients with UH and vice versa, assessed by clinical examination and abdominal ultrasonography at enrollment. Secondary outcomes included the identification of independent risk factors for coexistence using multivariate logistic regression based on variables such as sex, BMI, age, comorbidities, tobacco use, and obstetric history. Optimal BMI thresholds predictive of each condition were determined through ROC curve analysis. Gender-based prevalence and subgroup BMI distributions were also compared. All outcomes were prospectively assessed using standardized protocols across participating centers.

Statistical analysis

All statistical analyses were performed using SPSS for Windows, version 22.0 (IBM Corp., Armonk, NY). The Kolmogorov-Smirnov and Shapiro-Wilk tests were applied to assess data normality. Non-normally distributed variables were analyzed using non-parametric methods. Continuous variables were compared using the Mann-Whitney U test, while categorical variables were analyzed using chi-square or Fisher’s exact tests, as appropriate. Univariate logistic regression was used to identify potential risk factors for cholelithiasis in the UH group and umbilical hernia in the CL group. Variables with a p-value ≤ 0.25 in univariate analysis were included in a multivariate logistic regression model. Results were reported as odds ratios (ORs) with 95% confidence intervals (CIs) and corresponding p-values. Receiver operating characteristic (ROC) curve analysis was conducted to evaluate the predictive value of BMI for each condition. The area under the curve (AUC), 95% CI, optimal cut-off values (determined using the Youden index), sensitivity, and specificity were calculated. Categorical variables were expressed as frequencies and percentages, and continuous variables as means, medians, and ranges. A p-value < 0.05 was considered statistically significant.

## Results

**Figure 1:**
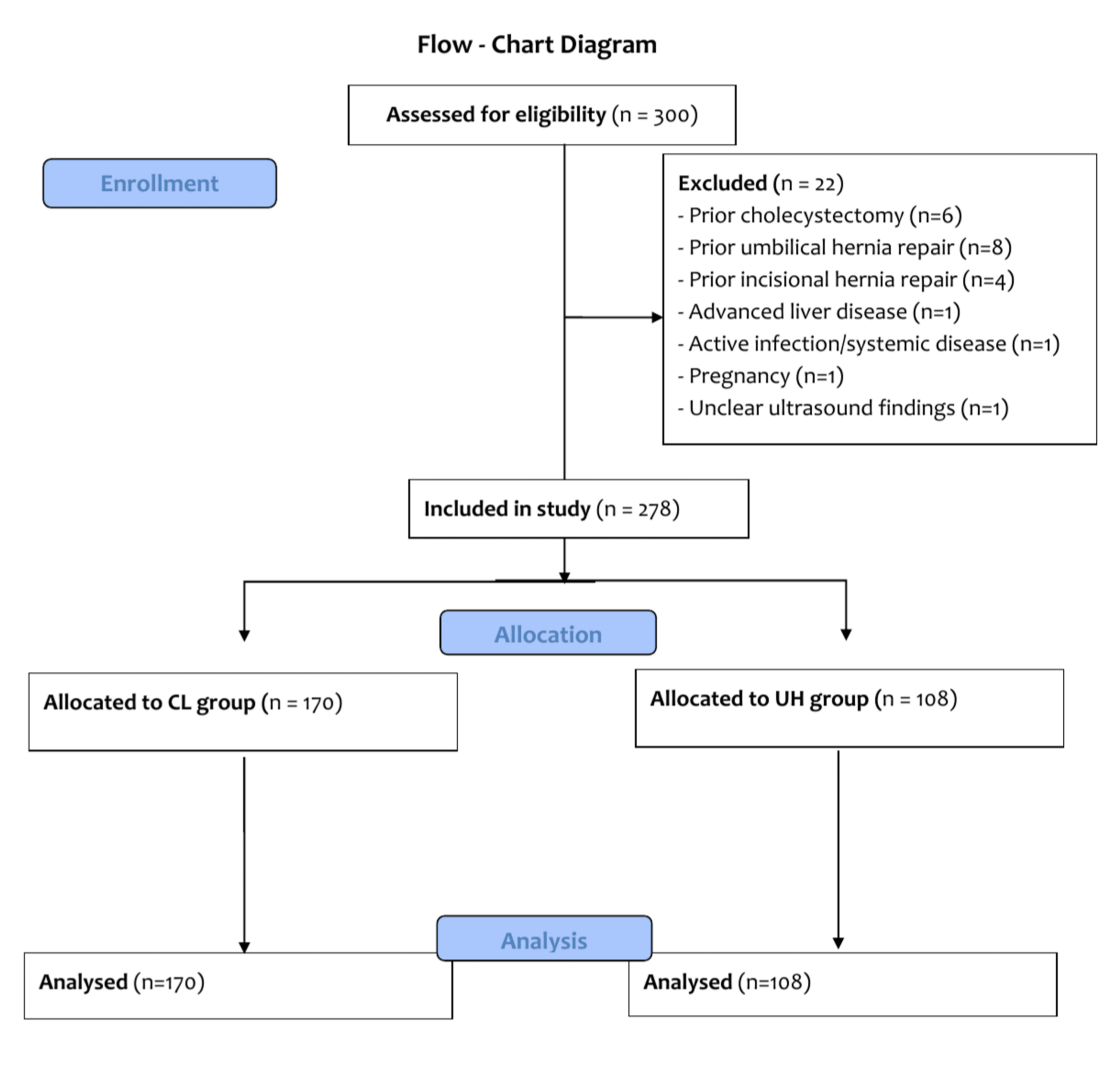
CONSORT Flow Diagram This diagram summarizes patient enrollment, exclusion criteria, group allocation, and analysis.

The study included 278 patients, with 170 allocated to the CL group and 108 to the UH group after applying exclusion criteria. The detailed patient selection process and exclusions are illustrated in Figure 1.

**Figure 2:**
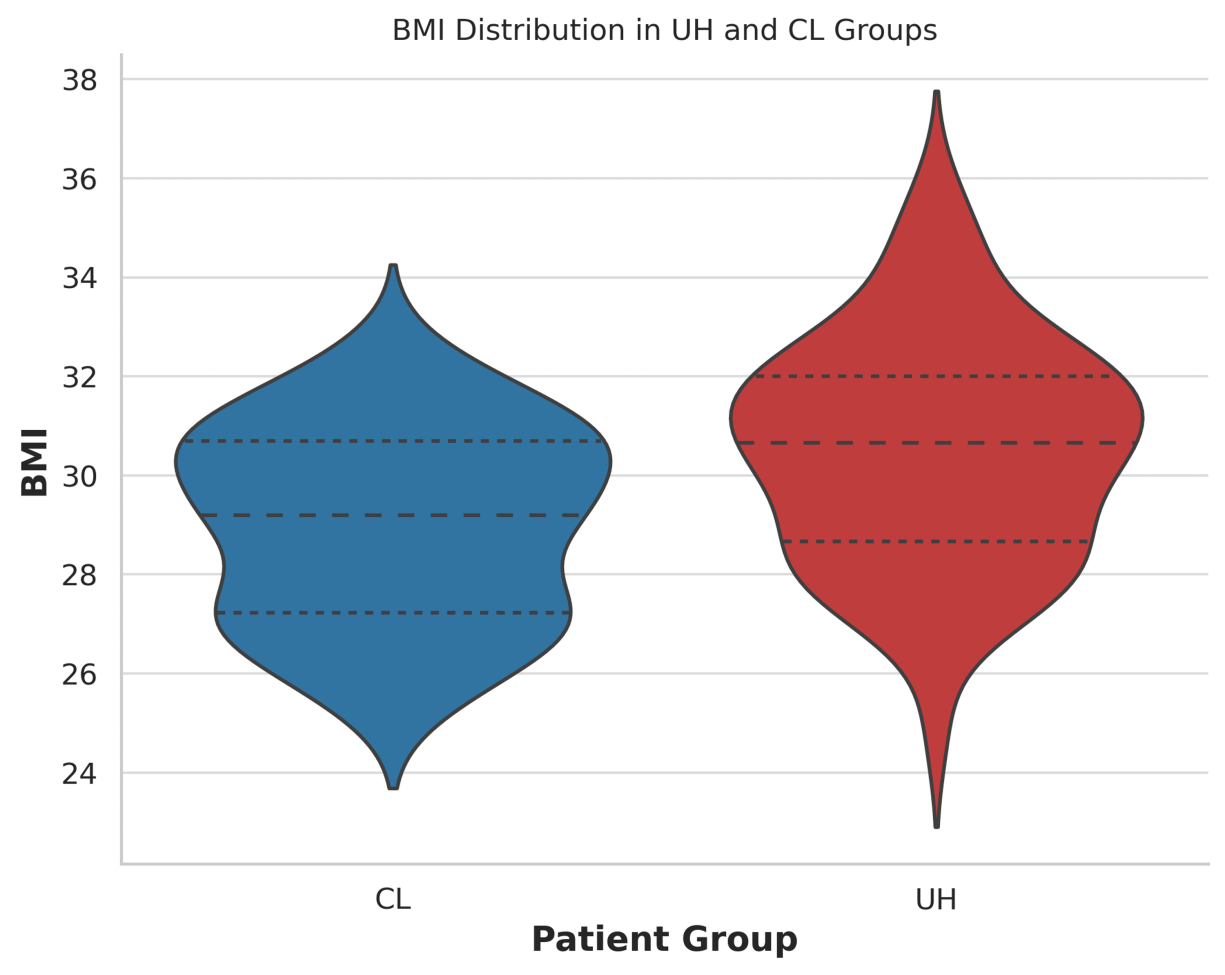
Violin plot of BMI distribution in UH and CL groups. This violin plot shows the distribution of BMI values in patients with UH and CL. UH, umbilical hernia; CL, cholelithiasis

The demographic characteristics and clinical features of the study population are summarized in Table 1. The median age was significantly higher in the UH group (53.5 years, IQR 41.25-65) compared to the CL group (46.5 years, IQR 38-57.25) (p = 0.03). In the CL group, 110 (64.7%) were female and 60 (35.3%) male; in the UH group, 66 (61.1%) were female and 42 (38.9%) male (p = 0.26). Median BMI was 28.3 kg/m² (IQR 26.7-30.4) in the CL group and 29.4 kg/m² (IQR 26.7-33.1) in the UH group (p = 0.09). The violin plot shows the BMI distribution in the UH and CL groups (Figure 2).

**Table 1:** Demographic variables and clinical features Categorical variables are shown as n (%), and continuous variables as median (Q1–Q3). Chi-square and Mann–Whitney U tests were used, with p < 0.05 considered significant. BMI, body mass index; UH, umblical hernia; CL, cholelithiasis; mm, millimeter; U, Mann–Whitney, U; x², chi-square.

Item	CL Group (n= 170)	UH Group (n= 108)	Test Statistics	p-value
Age	46.5 (38-57.25)	53.5 (41.25-65)	U = 7265.5	0.03
Gender (female/male)	110 (64.7%) / 60 (35.3%)	66 (61.1%) /42 (38.9%)	x² = 0.97	0.26
BMI	28.3 (26.7-30.4)	29.4 (26.7-33.1)	U = 7786.5	0.09
Smoking	59 (34.7%)	45 (41.6%)	x² = 0.54	0.39
Concomitant disease	50 (29.4%)	56 (51.9%)	x² = 13.16	<0.001
Family history	29 (17.1%)	21 (19.4%)	x² = 0.12	0.61
Gave birth	110 (64.73%)	53 (49.1%)	x² = 1.16	0.28
Multiple pregnancies	5(2.9%)	0 (0%)	x² = 1.49	0.22
Umbilical defect size (mm)	13.5 (10-17)	18 (12-22)	U = 678.0	<0.001

The proportion of smokers was 34.7% in the CL group and 41.6% in the UH group (p = 0.39). Comorbidities (diabetes, hypertension, metabolic syndrome) were more common in UH patients (51.9%) than in CL patients (29.4%) (p < 0.001). The umbilical defect size was larger in the UH group (18 mm) compared to the CL group (13.5 mm, p < 0.001; Table 1). The prevalence of UH in the CL group was 25.9%, while the prevalence of CL in the UH group was 29.6% (Table 2). Among CL patients with UH, the male-to-female ratio was 5:39 (p < 0.001). Among UH patients with CL, the male-to-female ratio was 7:25 (p < 0.001; Table 2).

**Table 2:** Comparison of the frequency of UH in CL patients and CL frequencies in UH patients by gender Chi-square test was used for comparisons; data are presented as frequency and percentage. A p-value of <0.05 was considered statistically significant. CL, cholelithiasis; UH, umbilical hernia

Items	(Cases/n)	Male	Female	Test statistic (χ²)	p-value
Frequency of UH in CL patients	44/170	5 (2.9%)	39 (23%)	χ² = 21.57	<0.001
Frequency of CL in UH patients	32/108	7 (6.5%)	25 (23.1%)	χ² = 8.17	<0.001

In the univariate analysis, female sex (p = 0.004), obesity (BMI ≥ 30 kg/m², p = 0.031), and concomitant disease (p = 0.015) were identified as significant risk factors for CL in the UH group (Table 3). However, in the multivariate logistic regression model, only female sex remained an independent predictor for CL in UH patients (OR: 0.221, 95% CI: 0.068-0.724, p = 0.013). In the CL group, female sex (p = 0.005), obesity (BMI ≥ 30 kg/m², p = 0.001), and a history of childbirth (p = 0.002) were significant risk factors for UH in the univariate analysis (Table 3). Multivariate analysis demonstrated that only obesity remained an independent predictor for UH in CL patients (OR: 3.443, 95% CI: 1.337-8.886, p = 0.01). 

**Table 3:** Logistic regression analysis of risk factors for cholelithiasis in umbilical hernia patients and for umbilical hernia in cholelithiasis patients. OR, odds ratio; CI, confidence interval; CL, cholelithiasis; UH, umbilical hernia *Included in the multivariate logistic regression model.

Risk factors	Univariate analysis for CL in UH		Multivariate analysis for CL in UH		Univariate analysis for UH in CL		Multivariate analysis for UH in CL	
	OR (95% CI)	p-value	OR (95% CI)	p-value	OR (95% CI)	p-value	OR (95% CI)	p-value
Female sex	0.181 (0.057–0.576)	0.004*	0.221 (0.068–0.724)	0.013	0.195 (0.063–0.604)	0.005*	0.311 (0.027–3.596)	0.349
Currently smoking	0.539 (0.218–1.336)	0.182	-	-	0.539 (0.218–1.336)	0.182	-	-
Age >40	1.745 (0.528-5.772)	0.361	-	-	1.451(0.559-3.767)	0.445	-	-
Obesity (BMI >30 kg/m2)	2.917 (1.102-7.723)	0.031*	1.913 (0.651–5.624)	0.238	4.412 (1.814-10.727)	0.001*	3.443 (1.337–8.886)	0.01
Gave birth	1.880 (0.777–4.551)	0.161	-	-	5.400 (1.898–15.365)	0.002*	1.658 (0.170–16.133)	0.663
Concomitant disease	3.115 (1.252–7.751)	0.015*	2.229 (0.811–6.123)	0.12	1.818 (0.733–4.511)	0.197	-	

**Figure 3:**
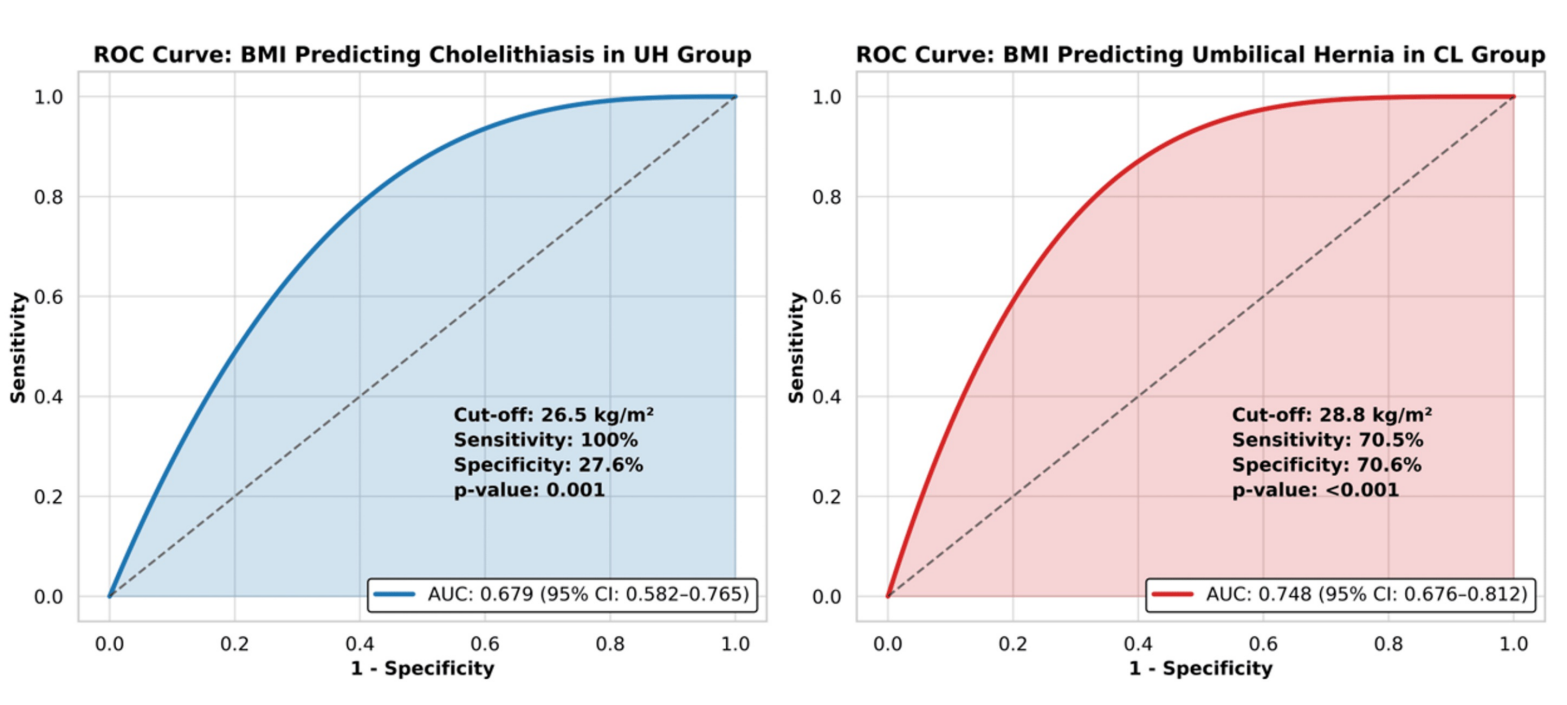
ROC Curves for BMI Predicting CL and UH ROC curves of BMI for predicting cholelithiasis in the UH group and umbilical hernia in the CL group. ROC, receiver operating characteristic; CL, cholelithiasis; UH, umbilical hernia; AUC, area under the curve; CI, confidence interval

Receiver operating characteristic (ROC) curve analysis was conducted to evaluate the predictive value of BMI for CL in the UH group and for UH in the CL group (Figure 3). The optimal BMI cut-off value for predicting CL in the UH group was 26.5 kg/m², with an AUC of 0.679 (95% CI: 0.582-0.765, p = 0.001), a sensitivity of 100%, and a specificity of 27.6%. The BMI cut-off value for predicting UH in the CL group was 28.8 kg/m², with an AUC of 0.748 (95% CI: 0.676-0.812, p < 0.001), a sensitivity of 70.5%, and a specificity of 70.6% (Table 4).

**Table 4:** ROC analysis for high BMI in predicting umbilical hernia and cholelithiasis in CL and UH groups. ROC, receiver operating characteristic; CL, cholelithiasis; UH, umbilical hernia; AUC, area under the curve; CI, confidence interval

Items	UH group	CL group
	Cholelithiasis predicting	Umbilical hernia predicting
AUC (95% CI)	0.679 (0.582–0.765)	0.748 (0.676-0.812)
Standard deviation	0.054	0.044
Cut-off value	26.5	28.8
Sensitivity	100%	70.50%
Specificity	27.60%	70.60%
P-value	0.001	<0.001

## Discussion

The coexistence of CL and UH has important clinical and surgical implications. Overlooking CL during UH repair may lead to technical difficulties in a subsequent minimally invasive cholecystectomy due to fibrotic changes and adhesions. Similarly, an unnoticed umbilical hernia can result in intra-abdominal organ injury during optical port insertion, and the risk of port-site hernia may increase following laparoscopic cholecystectomy [12,17].

The first documented association between umbilical hernia and cholelithiasis was reported in 1963 in the Argentinian medical journal "El Día Médico" [17]. Subsequently, in 1969, Bryant and Griffen highlighted this relationship in a series where 40% of patients with umbilical hernia also had gallbladder disease, drawing attention to the predominance of female, overweight, and multiparous patients in this group [8]. In line with these earlier observations, our study demonstrated that the prevalence of UH in CL patients and the prevalence of CL in UH patients were significantly higher in female patients (Table 2).

Several retrospective studies have attempted to quantify the coexistence of these conditions, reporting simultaneous umbilical hernia in 0.9% to 10.5% of cholelithiasis patients [18-26]. However, these studies primarily focused on the frequency of simultaneous umbilical hernia repair and cholecystectomy rather than investigating the actual prevalence of umbilical hernia in CL patients or vice versa. A recent retrospective study by Bozkırlı et al. reported that 23% of patients undergoing cholecystectomy also had umbilical hernia, with significantly higher BMI values in these patients compared to those without hernia (32.6 vs. 28.1 kg/m²) [10]. Similarly, our study found that 25.9% of CL patients had UH, and obesity (BMI ≥ 30 kg/m²) was the only independent predictor of UH in CL patients with a cut-off value of 28.8 kg/m². The BMI cut-off for predicting cholelithiasis in UH patients showed 100% sensitivity but low specificity (27.6%), indicating a high false-positive rate. This may reflect potential clinical relevance, overfitting, or sample-specific factors. Therefore, the result should be interpreted cautiously and confirmed in larger, independent cohorts.

The prevalence of cholelithiasis in patients diagnosed with umbilical hernia has been less frequently studied. Enshaie et al. investigated patients with various anterior abdominal wall hernias using ultrasound screening and detected cholelithiasis in 13.8% of UH patients [27]. Our study, however, found that 29.6% of UH patients had cholelithiasis, which is more than double the prevalence reported by Enshaie et al.

To properly contextualize our findings, it is essential to compare them with general population data. Epidemiological studies on umbilical hernia prevalence are limited. Bedewi et al. reported that 24.9% of women and 23.3% of men had an umbilical hernia when screened via ultrasound [15], whereas a study by Sazhin et al. in a Russian population found lower prevalence rates of 12.7% in men and 8.7% in women based on physical examination [16]. In our study, the prevalence of umbilical hernia in CL patients was 25.9%, which is comparable to the rates reported by Bedewi et al. but significantly higher than those found by Sazhin et al.

Similarly, the reported prevalence of cholelithiasis in the general population varies widely. A review by Acalovschi analyzing nearly 30 studies found that cholelithiasis prevalence ranges from 2.3% to 20.3% in men and 3.7% to 37.4% in women [23]. Large-scale European studies have reported prevalence rates of 8.2% to 9.5% in men and 18.0% to 18.9% in women [24,25]. In contrast, our study found a significantly higher cholelithiasis prevalence (29.6%) in UH patients, suggesting a potential association beyond chance. Given that many gallstones remain asymptomatic and are detected incidentally, determining their true prevalence remains challenging.

Beyond epidemiological associations, some researchers have explored the underlying pathophysiology linking cholelithiasis and hernias. Yamanaka et al. proposed a possible "tetralogy" by adding umbilical hernia to Saint’s triad (hiatal hernia, diverticulosis coli, and cholelithiasis) [28]. Hauer-Jensen et al. examined over 637,000 patient records and hypothesized that herniosis, a systemic connective tissue disorder, might be a shared etiology for Saint’s triad and abdominal wall hernias [29]. While their findings support a role for collagen metabolism disorders in hernia formation, a direct link to cholelithiasis remains unclear. Future research investigating collagen structure in the gallbladder wall, in addition to connective tissue analysis in hernia patients, may provide further insights into this potential shared etiology [26-29].

This study has some limitations. Its cross-sectional design prevents establishing a causal relationship between obesity, cholelithiasis, and umbilical hernia. While these conditions are associated, whether obesity directly contributes or other metabolic/connective tissue factors are involved remains unclear. Additionally, as the study population consisted of elective general surgery patients, the findings may not fully represent the general population.

Investigating collagen metabolism in cholelithiasis patients with and without umbilical hernias may clarify the role of connective tissue abnormalities. Larger prospective studies with diverse populations are needed to determine whether obesity and connective tissue disorders are key factors in this association.

Given that most gallstones are asymptomatic, as observed in the UH group in our study, elective cholecystectomy is generally not recommended unless patients become symptomatic [30]. However, considering the high coexistence rate, patients presenting with umbilical hernia should be screened for cholelithiasis using hepatobiliary ultrasonography, even in the absence of symptoms. If mesh herniorrhaphy has been performed and subsequent cholecystectomy is required, port placement should be carefully planned to avoid previous surgical sites and adhesions.

## Conclusions

The coexistence of cholelithiasis and umbilical hernia appears relatively common. Ultrasound screening for cholelithiasis may be considered in UH patients with elevated BMI or other risk factors to assist surgical planning and avoid adhesion-related challenges during potential future cholecystectomy. As both conditions may be asymptomatic, management should be individualized based on patient-specific factors and surgical risk.
